# Genome-Wide Screening for MYB Transcription Factors Involved in Flavonoid Glycoside Biosynthesis in *Carthamus tinctorius* L.

**DOI:** 10.3390/genes16111376

**Published:** 2025-11-11

**Authors:** Xiaohan Yu, Bin Xian, Lijun Peng, Xunjian Wu, Juncheng Zhang, Yuanyuan Li, Yueying Hu, Jiang Chen

**Affiliations:** 1 School of Pharmacy, Chengdu University of Traditional Chinese Medicine, Chengdu 611731, China; 15610790932@163.com (X.Y.); xianbin@stu.cdutcm.edu.cn (B.X.); penglj2025@163.com (L.P.); wuxunjian1@stu.cdutcm.edu.cn (X.W.); z1307098823@163.com (J.Z.); liyuanyuan0420777@163.com (Y.L.); yueyinhuy@163.com (Y.H.); 2 Key Laboratory of Standardization of Chinese Medicine, Chengdu University of Traditional Chinese Medicine, Ministry of Education, Chengdu 611731, China; 3 Lab for Innovation & Effective Uses of Chinese Drug Germplasm Resources, Chengdu 611731, China

**Keywords:** safflower, MYB, expression patterns, transcription factor, MeJA, flavonoid glycosides

## Abstract

Background: Safflower (*Carthamus tinctorius* L.) is a multipurpose crop with both medicinal and economic values. Flavonoid glycosides are the core bioactive components of this species for preventing and treating cardiovascular and cerebrovascular diseases, yet their specific regulatory mechanisms remain insufficiently systematically elucidated. Methods: Based on the whole-genome data of *Carthamus tinctorius* L., key MYB transcription factors regulating the flavonoid glycoside biosynthesis pathway in safflower were screened and verified via MeJA treatment. Results: A total of 202 MYB transcription factors were identified, and 18 candidate genes were screened out. Further analysis showed that four genes (HH_019113, HH_009268, HH_009443 and HH_029380) were extremely significantly positively correlated with flavonid glycoside biosynthesis genes. After MeJA treatment, RT-qPCR analysis showed that their expression levels were significantly different. Conclusions: With the objective of elucidating the biosynthesis mechanism of flavonoid glycosides in safflower and exploring key regulatory genes, this study identified four MYB transcription factors that regulate flavonoid glycoside biosynthesis, providing new insights into elucidating the biosynthesis mechanism of flavonoid glycosides in safflower and offering targets for the construction of its molecular regulatory network and the improvement of medicinal quality and molecular breeding technology

## 1. Introduction

Safflower (*Carthamus tinctorius* L.), belonging to the Asteraceae family, is a multi-purpose crop with both medicinal and economic value. Its medicinal part is the dried tubular flower, which, according to traditional Chinese medicine theory, has the effects of promoting blood circulation to remove blood stasis and dredging meridians to relieve pain, and is widely used in the prevention and treatment of Cardio-Cerebral Vascular diseases in clinical practice. Modern pharmacological studies have confirmed that flavonoids are the characteristic active ingredients and the material basis for the efficacy of safflower, among which flavonols (such as kaempferol and quercetin) and chalcones (such as HYSA) are the main pharmacodynamic components [1]. These flavonoids not only exhibit diverse pharmacological activities, including anti-oxidative stress, anti-inflammation, and inhibition of platelet aggregation, but also play a core role in the prevention and treatment of Cardio-Cerebral Vascular diseases through pathways such as improving microcirculatory disorders and regulating lipid metabolism [2,3]. However, a thorough and comprehensive analysis of the precise mechanism underlying these flavonoid components with high pharmacological value—particularly flavonoid glycosides—in safflower plants has not yet been conducted. This has limited our ability to fully comprehend how the therapeutic properties of safflower are formed.

The majority of research on the biosynthesis pathway of flavonoid glycosides in safflowers is based on the universal pathway of plant flavonoid biosynthesis, which is derived from the phenylalanine metabolic pathway, combined with transcriptomics, metabolomics and functional verification experiments. Our research team has previously sorted out several important enzyme genes and regulatory factors that may be involved in the synthesis of glycosides from safflowers. Ren et al. (2024), for instance, discovered that CtUGT3 correlated positively with astragalin accumulation, demonstrating its central function in the synthesis of flavonol glycosides [4]. Through genome-wide screening and expression profile analysis, Xian et al. (2024) discovered *OGT1*, a crucial gene involved in the synthesis of flavonoid 7-O-glycosides in safflower [5]. Xi et al. (2025) screened multi-omics data and discovered UGT95A2 with flavonoid 3′-O-glycosylation activity for the first time [6]. The regulatory network for the biosynthesis of safflower flavonoid glycosides is, therefore, lacking since previous research has only mined and validated the important enzyme genes in the biosynthetic pathway and has not thoroughly investigated the upstream transcription factors that control the expression of these enzyme genes. MYBs are extensively involved in flavonoid biosynthesis. Moreover, MYB-UGT regulatory networks have been identified in other species such as *Arabidopsis thaliana* and tea plants. Screening *MYB* genes is crucial for elucidating the regulatory mechanisms of safflower flavonoid glycosides.

Genome-wide screening technology has emerged as a key technique for extracting transcription factors in research for the control of plant secondary metabolism. Hormone therapy and gene editing to alter expression are two techniques for screening transcription factors. Since it can precisely forecast the regulatory interactions between transcription factors and target genes based on the correlation of gene expression patterns, co-expression analysis stands out among these as having indispensable advantages in the mining of functional transcription factors. As a result, it has been extensively used in studies of secondary metabolism in a variety of plant species. By building gene co-expression networks of leaves and shoot tips, researchers were able to successfully screen transcription factors from the MYB and bHLH families in studies on the regulation of flavonoids in *Camellia sinensis* (tea plant) [7]. Through co-expression correlation research, CsMYB86 and CsbHLH35 were specifically identified as important regulatory factors. These two factors can bind to the promoter regions of important genes for flavonoid biosynthesis enzymes (CHS, F3H) in a synergistic manner, greatly accelerating the accumulation of catechins. Co-expression analysis is also essential in research on the formation of phytoalexins (such as momilactone A) in *O. sativa* (rice). In order to test the WRKY family transcription factor OsWRKY71, researchers examined gene expression profiles under rice blast (Magnaporthe oryzae) stress. A primary objective for figuring out the regulation network of rice disease-resistant secondary metabolism is its co-expression relationship with important genes involved in phytoalexin production (OsCPS4, OsKSL4) [8]. Virág et al. discovered that hormonal signaling networks dynamically reorganize during developmental timing, with distinct hormone-dominant regulatory mechanisms at different stages. They identified 45 expressed MADS-box transcription factors, revealing their potential reproductive developmental functions and providing molecular targets for improving flowering timing and inflorescence structure in soybeans [9]. However, research on the control of flavonoid glycoside production in safflowers has not completely utilized these tried-and-true transcription factor screening techniques, particularly co-expression analysis. A reference model for examining the regulatory processes of safflower flavonoid glycosides is lacking due to the paucity of cases involving the mining of transcription factors specific to these distinctive components in safflower.

Research on transcription factors from families including WRKY and MYB in safflower has advanced. There is a research vacuum because the majority of the studies that are now available rely on expression profile screening and concentrate on the general biosynthesis of flavonoids, giving little consideration to the regulatory aspects of flavonoid glycosides [10,11,12]. In a systematic study of the R2R3-MYB transcription factor family in the safflower genome, for example, Liang et al. discovered CtMYB76, a crucial regulatory factor for the production of safflower flavonols. The expression of important genes involved in flavonoid biosynthesis can be activated by this factor’s direct binding to their promoters. To close this gap, this study integrates transcriptomic results under different conditions, phenotypic and metabolic features of various tissues, and safflower genome-wide data. The purpose of this integration is to identify key MYB transcription factor members involved in the regulation of flavonoid glycoside biosynthesis. It further conducts an in-depth analysis of the correlation between their expression patterns (in different tissues and under different environmental conditions) and the accumulation of flavonoid components. This study also investigates the intrinsic mechanism by which MYB transcription factors contribute to the biosynthesis of flavonoid glycosides through co-expression analysis. The objective of this study is to establish a foundation for the genetic enhancement of safflower’s medicinal quality and to offer a fresh theoretical framework for clarifying the biosynthetic regulatory network of safflower flavonoid glycosides.

## 2. Materials and Methods

### 2.1. Identification of MYBs in Safflower Genome

The safflower genome data were obtained from our previous study, and gene annotation was already performed [13]. The genomic sequence and annotation information of safflower are available at the National Genomics Data Center (NGDC) with the project accession number PRJCA009936 and genome accession number GWHBJIR00000000. All RNA-seq data have been deposited in the Sequence Read Archive (SRA) (http://www.ncbi.nlm.nih.gov/, accessed on 8 April 2025) under the accession number SUB13799758. We retrieved all MYB-related sequences characterized by the Pfam domain PF00249. The following procedures were carried out using The Arabidopsis Information Resource (TAIR, http://www.arabidopsis.org/, accessed on 8 April 2025) in order to extract *MYB* family genes from *A. thaliana*. The physical and chemical properties of MYB proteins were analyzed using the ExPASy (Expasy—ProtParam tool) (https://web.expasy.org/protparam/, accessed on 12 April 2025) online software. The amino acid sequences of 202 *MYB* genes were submitted to the ProtParam tool to calculate various parameters, including amino acid count, molecular weight, theoretical isoelectric point, instability index, aliphatic index, and grand average hydropathicity.

### 2.2. Evolutionary Classification and Phylogenetic Analysis of CtMYB Gene Family

To investigate the evolutionary relationships among members of the *CtMYB* gene family, a phylogenetic tree was constructed using MEGA 12 (V12.0.9) software (https://www.megasoftware.net/, accessed on 17 August 2025). The MUSCLE algorithm was used to align the protein sequences of the *MYB* genes (default gap open = −2.90, extend = 0, Hydrophobicity Multiplier = 1.20). TBtools software’s trimAl function was used to automatically trim regions with very low similarity. The phylogenetic tree was then created using the maximum likelihood (ML) approach, and the dependability of the branching patterns was evaluated by computing bootstrap values using 5000 repeats. The model is JTT, with Uniform Rates selected for Sites and All Points for Gaps/Missing Data. Lastly, the web tool iTOL (https://itol.embl.de, accessed on 19 August 2025) [14] was used to annotate and show the phylogenetic tree that had been built.

### 2.3. Conserved Motif Analysis, Gene Structures, and Chromosomal Mapping of MYBs

Conserved motif analysis results of *MYB* genes were obtained using MEME (V5.5.8) (https://meme-suite.org/meme/tools/meme, accessed on 11 November 2025) [15], with the number of motifs set to 15 after many experiments and comparisons; the minimum motif width was set to 6, and the maximum motif width was set to 50, with E-value < 0.05 and *p*-value < 0.0001. The gene structure and chromosomal localization of *MYB* genes were determined based on the GFF file of the safflower genome and visualized using TBtools (V2.345) (https://tbtools.cowtransfer.com/s/0a9cbf41b47b4a, accessed on 20 October 2025) [16].

### 2.4. Cis-Acting Elements Analysis in the Promoter Region of MYB

To better analyze the transcriptional regulation mechanism of *MYB* genes, the 2000 bp sequences located upstream of the start codon (ATG) of each transcript were extracted using tbtools. The cis-acting elements were studied with the assistance of the plantcare website (https://bioinformatics.psb.ugent.be/webtools/plantcare/html/, accessed on 20 October 2025) and the analysis results were visualized using TBtools (V2.345).

### 2.5. Transcriptome Analysis of Safflower

Data on different parts, different light intensities, and different flowering stages were all derived from previous studies. This data involves four key flowering stages, which were chosen during the flowering period. The four stages correspond to the 1st, 2nd, 3rd, and 4th days after flowering, respectively. Three samples were taken for each tissue at each stage, including roots, stems, leaves, and flowers (with three biological replicates for each sample). All samples were promptly frozen in liquid nitrogen.in order to perform RNA extraction and expression analysis later on.

In addition, the different light intensities include low light intensity (LL; 10,000 Lux), medium light intensity (ML: 20,000 Lux), and high light intensity (HL: 40,000 Lux). The expression data of MYB genes were extracted from the transcriptome data of these earlier investigations and transformed into FPKM values (fragments per kilobase of exon per million fragments mapped) for additional research. Cluster analysis was performed on the genes according to their expression levels. We used the Chiplot tool to create heatmaps, and an online program (http://www.cloud.biomicroclass.com, accessed on 16 July 2025) was used for categorization. Genes chosen from the intended clusters were subjected to Venn analysis using the web application (https://jvenn.toulouse.inrae.fr/app/example.html, accessed on 18 July 2025) [17].

### 2.6. Screening of MYB Transcription Factors Regulating Flavonoid Glycosides via Co-Expression Analysis

As light intensity increases, the flavonoid content of safflower initially rises and then falls, peaking three to four days after flowering. Thus, we used the website https://cloud.oebiotech.com/, (accessed on 1 November 2025) to undertake co-expression analysis after integrating the expression analysis data and screening prospective genes using Venn diagram analysis. Each set of samples was set with three biological repeats. The top 20 attributes were chosen for visual display after the Pearson correlation coefficient was calculated. The significance level was set at 0.05, and the correlation criterion between features was set at 0.8 in the network diagram [18]. The result graphs were filtered for additional analysis if the *p*-value was less than 0.001.

### 2.7. MeJA Treatment and qPCR Verification

The MeJA treatment protocol was developed based on the research methodology previously established by our research team [19]. Previous research indicates that MeJA is extensively involved in the biosynthesis of flavonoid glycosides. Safflowers were planted in Chengdu University of Traditional Chinese Medicine’s Wenjiang Campus Medicinal Plant Garden. The treatment group received a 100 μM methyl jasmonate (MeJA) solution sprayed on healthy safflower flowers three days after they flowered, while the control group received an equal volume of solution devoid of MeJA. Following treatment, the flowers were wrapped in clear plastic bags to facilitate solution absorption and stop the loss of volatile plant hormones. Six hours later, remove the bags to conduct sample collection, with three biological replicates set for each sample. Following sample collection, the samples were promptly frozen in liquid nitrogen and kept in a refrigerator set at −80 °C.

The qPCR verification stage involved reverse transcription using the Prime Script reverse transcription kit from Takara and total RNA extraction using the RNA extraction kit from Invitrogen. Each set of samples was set with three biological repeats. Primer 5.0 was used to build primers for the MYB gene, using the reference gene, the 28S coding region of safflower. Three repetitions were set up to confirm the expression level in the experiment, which was carried out using the Bio-Rad CFX96 real-time PCR detection system using the Takara SYBR Prime Script RT-PCR kit.

## 3. Results

### 3.1. Identification of MYBs in the Safflower Genome

We identified a total of 202 transcripts belonging to the *MYB* family. The protein length of MYB ranges from 118 to 1491 amino acids, with a molecular weight of 13.97–162.78 kDa and a Theoretical isoelectric point of 4.50–10.26. Subcellular localization prediction shows that most MYB proteins (191 out of 202) are located in the nucleus, and very few (7 out of 202) are located in chloroplasts. Other comprehensive details and characteristics, including the overall average values of instability index, aliphatic index, and hydrophilicity, are presented in Table S1.

### 3.2. Evolutionary Classification and Phylogenetic Analysis of Ct MYB Gene Family

**Figure 1:**
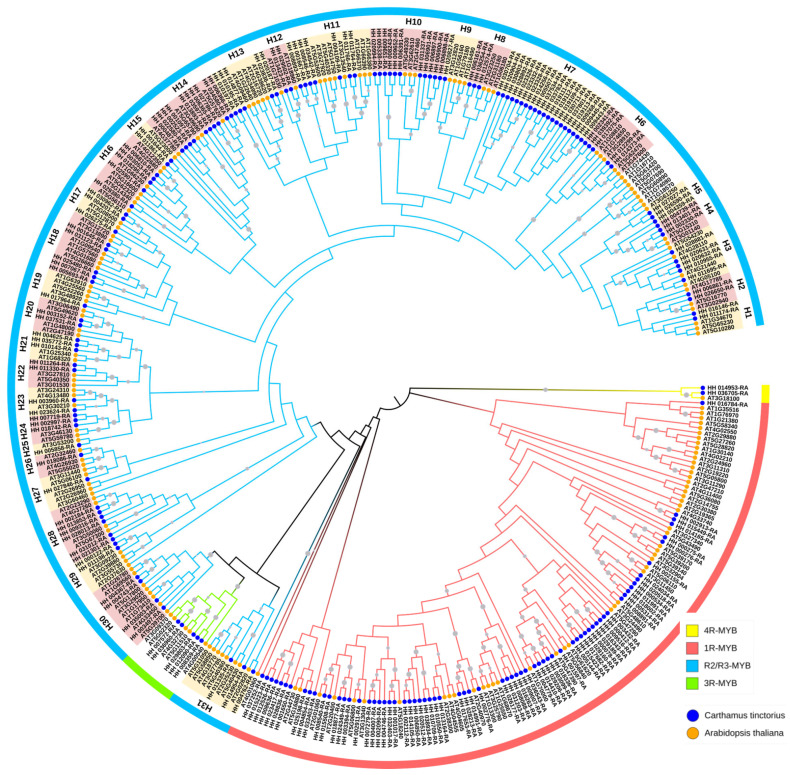
Phylogenetic analysis of *MYBs* in safflower. The gray circles at the branch nodes indicate a Bootstrap value greater than 0.7. The families of *MYBs* are indicated by outer colored bands, while different species are represented by distinct colored circles.

Using Mega12 software, multi-sequence alignment and phylogenetic tree construction were performed for 202 *CtMYB* genes and 168 Arabidopsis *MYB* genes. (Figure 1 and details can be viewed in Table S2). Based on their homologous genes in Arabidopsis, the *CtMYB* gene family was classified into four distinct groups: The R2/R3-MYB family of 125 genes, the 3R-MYB family of 5 genes, the 4R-MYB family of 2 genes, and the 1R family of 70 genes [20]. Meanwhile, according to the phylogenetic tree topology and the classification of the *MYB* superfamily in Arabidopsis, the R2/R3 family was divided into 31 groups (H1–H31). Among them, H9, H19, H25, H26, and H27 each contained only one member, representing the smallest clades, while H7, with 20 members, constituted the largest clade. Additionally, members of H7 did not cluster into any clades of Arabidopsis. This may be related to the adaptation of safflowers to specific environments or the formation of unique metabolic pathways during their long-term evolutionary process, providing important clues for identifying MYB transcription factors that specifically regulate flavonoid glycoside biosynthesis in safflower.

### 3.3. Conserved Motif Analysis, Gene Structures, and Chromosomal Mapping of MYBs

**Figure 2:**
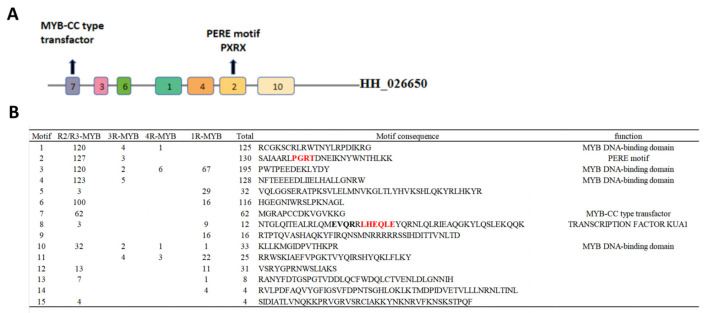
The distribution of conserved motifs and the detail of motif (**A**) *HH_026650* as an example, the schematic diagram of conserved motifs in safflower (*Carthamus tinctorius*) MYB proteins. (**B**) The distribution of 15 conserved motifs is shown. Characteristic motifs containing functional feature domains are labeled in the figure. Meanwhile, the exon distribution of *MYB* subgroup transcripts is presented. Red annotations indicate signature motifs containing domains with clear functional characteristics.

Conserved motif analysis revealed fifteen conserved motifs, with lengths up to 1500 amino acids, using MEME software (V5.5.8). Each CtMYB protein exhibited a unique combination of these motifs, indicating specific patterns within particular subgroups. (Figure 2A, and details can be viewed in Figures S1 and S2) Motifs 1, 2, 3, 4 and 6 show higher occurrence frequencies, potentially serving as conserved functional motifs. Notably, Motif 3 is present in each group, exhibiting a high level of conservation. Additionally, Motif 3 is often located at the C-terminus, while Motifs 2 and 10 are frequently found at the N-terminus. Overall, the distribution of these motifs exhibits significant differences across different families or groups, whereas similar patterns are observed within the same family or group. Motif 15 is exclusively present in the R2/R3-MYB family, while motif 9 occurs solely in the 1R-MYB family. Moreover, the vast majority of genes within the R2/R3 family exhibit a unique conserved sequence pattern of 7-3-6-1-4-2. As shown in Figure 2B, motif 1, motif 3, motif 4 and motif 10 were found to encode the MYB DNA-binding domain [21]. Motif 2 consisted of the PERE motif (PxRx). Motif 7 is a MYB-CC type transcription factor containing the LHEQLE motif. Motif 8 is TRANSCRIPTION FACTOR KUA1, which contains the Myb domain (PS51294), and its function tends to be related to stress regulation, while the other motifs did not have function annotation.

**Figure 3:**
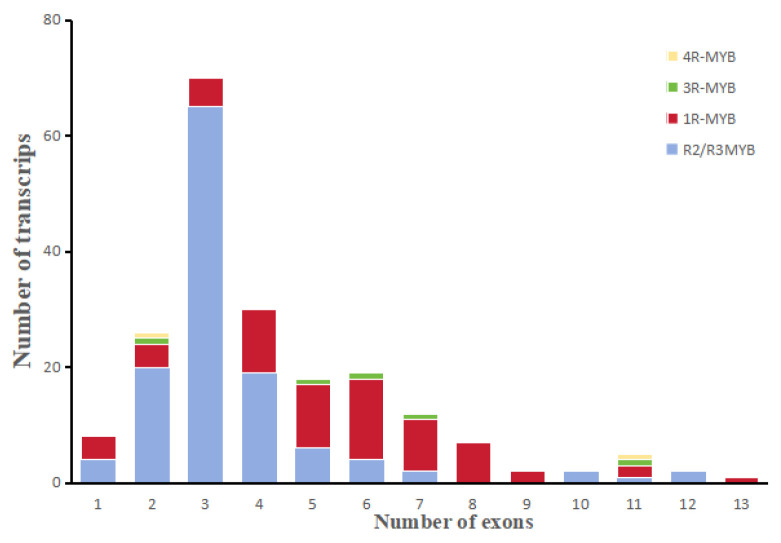
Exon distribution of MYB transcripts. Four colors correspond to different MYB family members.

Structural distribution of exons, introns, CDS, and UTRs of *MYB* genes was visualized and analyzed using TBtools to enhance the understanding of differences in evolutionary directions among different *MYB* subfamilies (Figure 3 and Figure S3). Most R2/R3-MYB genes contain 1–4 exons, while most 1R-MYB genes contain 5–8 exons, showing significant differences in quantity. The safflower genome annotation data was used to map the genetic locations on the chromosomes. The genome assembly comprises 1.17 Gb of sequence, distributed across 12 chromosomes (50.84–185.00 Mb) with an N50 of 96.39 Mb. 147 of the 202 MYB genes were dispersed at random throughout the 12 chromosomes. The remaining genes stayed anchored on scaffolds because they were unable to be mapped to particular chromosomes [22] (Figure S4). There was an uneven pattern in the chromosome distribution of *MYB* genes. With 42 genes, chromosome 1 had the highest density of *MYB* genes. On the other hand, with only four genes per chromosome, chromosomes 8, 10, and 11 had the smallest distributions of *MYB* genes.

### 3.4. Cis-Acting Elements Analysis in the Promoter Region of MYBs

**Figure 4:**
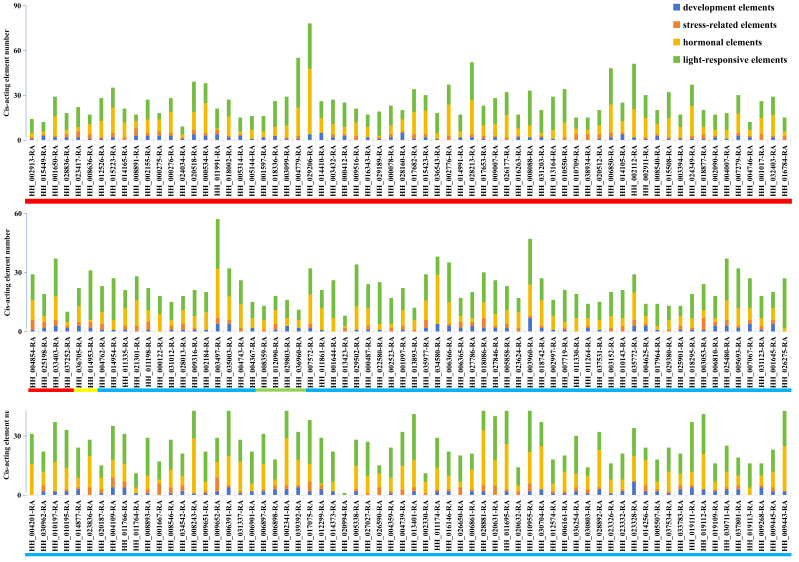
The predicted cis-acting elements are classified into four categories: developmental regulation, stress response, hormone regulation, and light response. Different colors below the image represent different subgroups of *MYB*.

A cis-acting element analysis was conducted, and the significantly expressed cis-acting elements identified in the prediction were involved in developmental regulation, hormone regulation, stress response, and light response (Figure 4, additional details in Figures S5 and S6). Among the predicted light-responsive elements, the four with the highest frequencies are G-Box (25.48%, 663 out of 2602), Box-4 (20.87%), GT1-motif (11.84%), and TCT-motif (6.96%), respectively. Followed by the hormonal-responsive cis-acting elements, ABRE has the highest occurrence frequency, accounting for 40.86%, and then CGTCA-motif and TGACG-motif, each accounting for 17.26%. Among the promoter development elements, 137 elements are related to protein metabolism regulation (39.48%), 71 elements are related to meristem expression (20.46%), 51 elements are related to the circadian rhythm (14.70%), and 28 elements are related to endosperm expression (8.07%). In addition, in safflower, there are also stress-related elements, among which LTR, TC-rich repeats, MBS, and GC-motif have relatively high occurrence frequencies, which are 37.01%, 27.16%, 25.37%, and 7.76%, respectively.

### 3.5. Expression Profiling of MYB Genes of Safflower Under Different Development Stages, Light Intensities and Tissues

There are several variables that control gene expression. According to promoter analysis, the main variables affecting *MYB* gene expression are hormone regulation, light, stress, and development. In early research, we discovered that the amounts of two kinds of flavonoid glycosides in safflower are strongly influenced by light intensity and developmental stage. Furthermore, there are significant differences in the kinds and concentrations of flavonoid glycosides in various tissue sections. The participation of *MYB* genes in the regulation network of flavonoid glycoside expression was further validated by cis-acting element analysis. Therefore, it will be easier to identify functional genes in future research if the expression patterns of *MYB* genes under different conditions are analyzed.

We examined the transcriptome data from safflowers in various tissues, light levels, and blooming stages (Figure S7). *MYB* genes were categorized into six groups based on their expression patterns; each cluster was made up of genes with comparable expression characteristics. 13–69 *MYB* genes were found in each cluster in the gene expression profiles under various light intensities (Figure S8A). Each cluster contained 23–37 *MYB* genes for the gene expression profiles throughout various embryonic stages (Figure S8B). There were 21–54 *MYB* genes in each cluster among samples from various organs (Figure S8C).

**[Different lighting treatments]** Cluster analysis under various light intensity treatments revealed that 79 *MYB* genes from clusters 2, 4, and 5 exhibited an expression trend that increased initially before dropping as light intensity increased. 39.24% of them were 1R-MYB, 56.96% were R2/R3-MYB, 2.53% were 3R-MYB, and 1.27% were 4R-MYB. **[Different flowering stages]** We concentrated on genes with an overall rising trend in expression levels for the cluster analysis of transcriptome data from the four flowering stages. A total of 90 *MYB* genes were found in clusters 3, 4, and 5, with 42.22% coming from 1R-MYB and 57.78% from R2/R3-MYB. **[Different tissues]** Since safflower flowers have the largest concentration of flavonoid components, we concentrated on clusters 4, 5, and 6, where 112 *MYB* genes were found [23]. The biggest percentage of these was R2/R3-MYB (58.93%), followed by 1R-MYB (36.61%), 3R-MYB, and 4R-MYB (3.57%), and 8.93%, respectively.

### 3.6. Screening of MYB Transcription Factors Regulating Flavonoid Glycosides via Co-Expression Analysis

**Figure 5:**
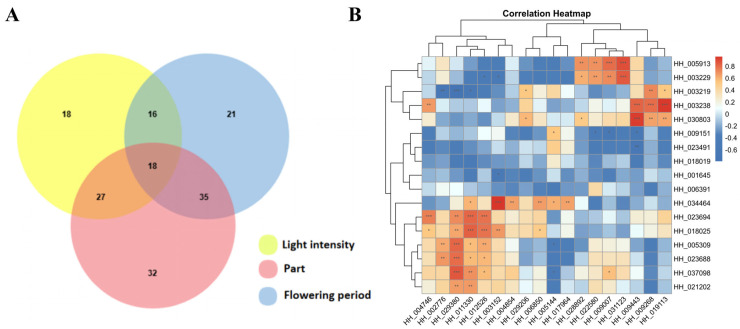
(**A**) Integrating three sets of transcriptome data for Venn diagram analysis to screen candidate genes. (**B**) Co-expression analysis to screen transcription factors that regulate flavonoid glycoside biosynthesis. * represent *p* < 0.05; ** represent *p* < 0.01; *** represent *p* < 0.001.

As light intensity increased, the flavonoid content of safflower first rose and then fell, peaking three to four days after flowering, according to expression analysis results. The spatiotemporal-specific biosynthesis of plant secondary metabolites is typically linked to specific spatiotemporal-specific biosynthetic pathway genes. In light of this, we combined the findings of the expression study under three different situations, chose gene clusters exhibiting comparable expression patterns for Venn diagram analysis, and ultimately discovered eighteen potential *MYB* genes (Figure 5A, and details can be viewed in Table S4).

Numerous enzymes and transcription factors linked to flavonoid production have been found in earlier research, and in this study, we combined metabolomic data to perform co-expression analysis (Figure 5B, and the visualization of the network diagram is shown in Figure S9). The analysis included 18 candidate genes and 17 functional genes, and 17 pairs of co-correlation were detected. The findings indicated that PAL1 and C3H1, important enzymes of the phenylpropanoid pathway, had a significant positive correlation with candidate genes like *HH_031123* and *HH_009007* (*p* < 0.05), suggesting that these candidate genes may be involved in the upstream phenylpropanoid metabolism process of flavonoid synthesis; UGT3, a key glycosyltransferase for flavonoid glycoside synthesis, had an extremely significant positive correlation with candidate genes like *HH_009268* and *HH_009443* (*p* < 0.001), suggesting that these candidate genes may synergistically regulate the biosynthesis of flavonoid glycosides alongside UGT. The glycosyltransferase OGT1 and several members of the MYB transcription factor family showed a highly significant positive correlation with *HH_029380* and other genes (*p* < 0.001), suggesting that this gene cluster may form a co-expression module to cooperatively participate in the synthesis of particular flavonoid glycosides. However, a few indicators showed significant negative correlation, and important enzymes for flavonoid synthesis, such as CHS3 and F3H3, mostly displayed a negative correlation trend with the majority of candidate genes. This suggests that there is little expression synergy between the majority of candidate genes and these enzymes. According to the results of the co-expression analysis, *HH_019113*, *HH_009268*, and *HH_009443* generally exhibited a highly significant positive correlation with the glycosyltransferase UGT3, which catalyzes the glycosylation of the 3-OH and 7-OH positions of the flavonol aglycone to directly produce astragalin. HH_029380 exhibited a highly significant positive correlation with OGT1, which can bind to the 7-OH site of flavonoid substrates and catalyze glycosyl transfer reactions to increase the production of flavonoid glycosides. These transcription factors may have distinct and significant functions in the regulation of the production of flavonoid glycoside components in safflowers, providing clues for the subsequent deciphering of the molecular regulatory network of flavonoid glycoside biosynthesis.

### 3.7. MYB Gene Expression Was Enhanced by MeJA Treatment

**Figure 6:**
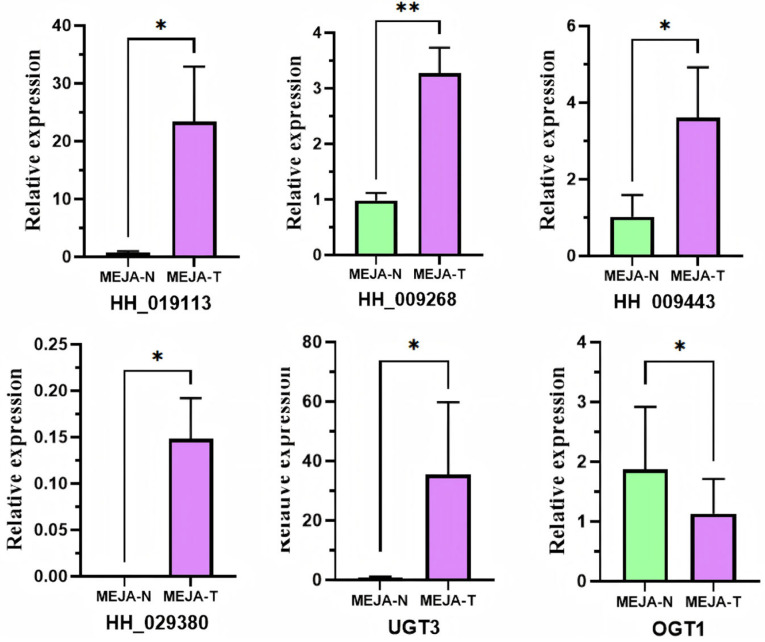
Expression under MeJA treatment. MeJA-N, no MeJA treatment; MeJA-T, MeJA treatment. Note: * represents *p* < 0.05, ** represents *p* < 0.01.

To verify the function of the screened key transcription factors involved in flavonoid glycoside biosynthesis in safflower, we treated them with MeJA and used qPCR to detect the expression dynamics of target genes in the treated samples (Figure 6). All data were validated using the T-test. Following MeJA treatment, the three MYB transcription factors linked to UGT3 expression (*HH_019113*, *HH_009268*, and *HH_009443*) demonstrated a consistent upregulation trend, with *HH_019113* showing the largest increase in expression, 23.34 times higher than the control group; *HH_009268* and *HH_009443* showed expression levels that were 3.27 and 3.61 times higher than the control group, respectively. Combined with the research group’s earlier findings, the amount of chalcone glycoside in safflower increased dramatically following MeJA treatment, and the total flavonoid glycosides exhibited a clear rising trend within 3–4 days of therapy. The experiment’s findings also demonstrated that these three MYB transcription factors linked to UGT expression can react to MeJA induction, regulate the expression of downstream important glycosyltransferase enzyme genes to contribute to the regulation of flavonoid glycoside biosynthesis in safflower, and that *HH_019113* may have a dominant regulatory role in this process.

In addition, *HH_029380*, which is linked to OGT1 expression, was also analyzed. The findings demonstrated that whereas natural flowers did not exhibit any *HH_029380* expression, MeJA treatment did result in a slight level of expression. Its expression pattern, however, differed from that of OGT1: following MeJA treatment, OGT1′s expression level dropped by 60.75%. This finding may suggest that *HH_029380* responds to MeJA in a unique way. It might not directly control OGT1 expression to contribute to the production of flavonoid glycosides, or it might require working in concert with other signaling pathways in certain spatiotemporal circumstances. Furthermore, it is not impossible that environmental influences or other unidentified metabolites have an impact on the control of this gene’s expression.

## 4. Discussion

In this study, a comprehensive analysis was conducted on the gene structure, protein motifs, and phylogenetic relationships of the safflower *MYB* family, and their subcellular localizations were predicted. We further investigated the expression patterns of *MYB* genes under abiotic stress treatments. Through systematic analysis of *MYB* gene expression patterns in different tissues, under varying light intensities, and at different developmental stages, this study provides key clues for in-depth exploration of the regulatory mechanisms of flavonoid glycoside compounds.

We identified a total of 202 safflower *MYB* genes and categorized them into four main groups based on their phylogenetic relationships. This number is fewer than that in crops such as *Oryza sativa* [24], *Zea mays* [25], *Glycine max* [26] and *Triticum aestivum* [27] but slightly more than that in crops like *Solanum lycopersicum* [28], *Cucurbita moschata* [29], and *Gossypium hirsutum* [30]. This quantitative difference is likely closely related to the evolutionary history, genome size, and biological characteristics of different plants [31]. Generally speaking, crops with larger genomes that have undergone multiple replication events tend to have a more abundant number of gene family members [32]. As a plant with specific medicinal value, the size of the *MYB* gene family in safflower may be adapted to its unique metabolic pathways and environmental adaptation strategies. In the future, through comparative analysis with the MYB gene families of more related species, it is expected to further reveal the evolutionary origin and functional differentiation characteristics of safflower MYB genes.

Through clustering analysis of CtMYB transcription factors and AtMYB members across multiple subgroups, high sequence conservation was observed within the same evolutionary subgroup. Based on the findings of Hong et al. [33] and Sukumaran [34], the high homology of these *CtMYB* genes is hypothesized to endow them with key functions similar to their Arabidopsis homologs. For example, the H1 subgroup regulates root system development, the H2 subgroup is critical for promoting low-temperature dormancy, and the H3 subgroup mediates responses to abiotic stresses, such as drought and salinity [35]. This conserved functional pattern across subgroups has also been reported in solanaceous and gramineous plants (Li et al., 2023) [36], indicating significant orthologous conservation of *MYB* subgroup functions during angiosperm evolution. R2/R3-MYB is the most abundant of the safflower *MYB* genes and performs important physiological and regulatory roles [37]. It clearly differs from other subfamilies in terms of conserved sequences and gene structure. The motifs 1, 2, 4, and 7, which are extensively found in R2/R3-MYB and are thought to have significant functions, are absent from the majority of non-R2/R3-MYB genes. In summary, the *MYB* gene family is extensively found in species with a variety of roles, and the structure’s variability allows for the development of different active ingredients in safflower. Understanding the crucial roles that *MYB* genes play in plant metabolic networks is made easier by their classification.

The cis-acting elements analysis confirms that the light-responsive promoter elements are the most abundant in the promoter of the safflower *MYB* gene family. Specifically, LTR is related to low temperature stress, TC-rich repeats are related to defense and stress responses, MBS is related to drought stress, and GC-motif is related to hypoxia induction. Among them, MBSI is involved in the regulation of flavonoid synthesis; AACA_motif, GCN4_motif [38,39,40], and others are involved in the regulation of endosperm synthesis. Additionally, there are elements related to stress responses such as drought, low temperature, and hypoxia, as well as plant hormones like gibberellin and abscisic acid, and light responses. The presence of these hormone-related cis-acting elements is consistent with the core mechanism revealed by Virág et al., wherein MYB transcription factors respond to hormone signals and regulate secondary metabolism by binding to specific cis-acting elements, suggesting that *MYB* genes in *Carthamus tinctorius* can integrate signal and metabolic regulation through hormone-cis-acting element interactions. Mutual regulation of *MYB* genes is demonstrated by the variations in the quantity and distribution of elements, such as deletions or repeats [41]. These results support the critical function that *MYB* genes play in safflower growth and development and can be used to enhance safflower cultivation practices in various environmental settings.

According to earlier research, safflower’s flavonoid glycoside content rises noticeably within one to three days or four days after flowering [42], and it varies with variations in light intensity. The discovery of numerous cis-acting elements linked to light, development, hormones, and stress by promoter analysis of *MYBs* further supports the idea that *MYB* genes are essential for the biosynthesis of flavonoid glycosides in safflower. These genes were categorized into various groups according to the ways in which *MYBs* expressed their genes under various circumstances. Meanwhile, the distribution of flavonoid glycosides in safflowers has clear tissue specificity, according to phenotypic and metabolic investigations of the roots, stems, leaves, and flowers. This study found 18 putatively important genes controlling the production of flavonoid glycosides by combining the findings of several gene clustering methods. Co-expression analysis was carried out using several previously identified enzymes and transcription factors involved in the biosynthesis of safflower flavonoid glycosides in conjunction with earlier research conducted by our research group. In particular, three potential MYB transcription factors that are downstream response components of the MeJA signaling pathway are co-expressed with UGT3 in safflower. By directly binding to cis-acting elements like light-responsive elements and hormone-responsive elements in the promoter region of the *UGT3* gene, these MYB transcription factors may further initiate the expression of UGT3. MeJA treatment can significantly activate their transcriptional expression. A positive regulatory chain of “MeJA-MYB TFs-UGT3-flavonoid glycosides” can be formed by activating the expression of UGT3, a crucial enzyme for flavonol aglycone glycosylation, which can encourage the manufacture of flavonoid glycosides such as astragalin. Furthermore, even though MeJA activation increases the expression of MYB transcription factors co-expressed with OGT1, their expression trend differs from that of OGT1. By creating a complex network of interactions with other transcription factors and signaling molecules, it is hypothesized that they indirectly contribute to the fine regulation of flavonoid glycoside biosynthesis rather than directly regulating OGT1. However, precise regulatory pathways require more investigation. The MYB-UGT regulation mode has also been investigated in safflower similar species, including *Camellia sinensis* [43,44] and *Arabidopsis thaliana* [45]. As a crucial regulator of flavonol biosynthesis in *Arabidopsis thaliana*, AtMYB12 can bind directly to MYB-binding elements in the promoters of glycosyltransferase and flavonol synthase (FLS) genes to activate their expression. In *Camellia sinensis*, CsMYB308 participates in the glycosylation modification of catechins by regulating UGT78A14. In this study, the regulatory mode of the MYB-UGT3 module in safflower shows conservation with these homologous systems, all reflecting the possible regulatory effect of MYB transcription factors on key glycosylation enzymes. However, the specific activation of this module by MeJA signaling in safflower suggests that it may have evolved unique regulatory nodes adapting to the accumulation of its own flavonoid glycosides. It is worth noting that this study only verified the existence of a certain correlation in the expression of transcription factors through MeJA induction, and the specific regulatory mechanisms require further in-depth investigation. Meanwhile, predictions based solely on co-correlation analysis have certain limitations. In the future, experimental techniques such as promoter fishing or yeast one-hybrid assay can be further combined for additional analysis and verification.

In safflower, the *MYB* gene family is essential for the production of flavonoid glycosides. This work found 202 *MYB* genes in safflower by combining genome-wide and multi-omics investigations. We then carried out analyses of the genes’ conserved motifs, gene structures, cis-acting elements, chromosomal placements, and phylogenetic relationships to acquire comprehensive gene family information. Additionally, we used cluster analysis on expression patterns across various developmental stages, light conditions, and tissue types to weed out candidate transcription factors. Lastly, we used co-expression analysis to identify important MYB transcription factors involved in the synthesis of flavonoid glycosides and carried out functional verification experiments. This study establishes the groundwork for further in-depth investigation and functional characterization while also offering fresh data for investigations into the production of flavonoid glycoside chemicals in safflower. In the future, we may achieve efficient enrichment of medicinal active components via the heterologous overexpression of identified MYB transcription factors or the construction of a “MYB-UGT/OGT” gene co-expression system to specifically enhance the synthesis pathway of safflower flavonoid glycosides. Alternatively, key MYB genes could serve as molecular markers when combined with marker-assisted breeding techniques, enabling rapid screening of superior single plants with a high flavonoid glycoside content.

## Data Availability

No new data were used in this study. The data used is already in the article.

## Supplementary Materials

The following supporting information can be downloaded at https://www.mdpi.com/article/10.3390/genes16111376/s1. Figure S1: Schematic diagram of conserved motifs in safflower MYBs, with different motifs represented by various colors. Figure S2: WebLogos of the 20 conserved motifs in safflower MYBs. Each letter in the WebLogos represents an amino acid, with its height indicating the relative frequency of that amino acid. Figure S3: Gene structures’ schematic diagram in safflower MYBs. Black and Purple rectangles denote untranslated regions (UTR) and coding sequences (CDS), respectively. Lines without rectangles represent introns. Figure S4: Safflower chromosome mapping based on genomic annotation data. Figure S5: Schematic diagram of four categories of cis-acting elements in the promoter of safflower MYBs. Figure S6: Heatmap display of the number of each cis-acting element in the promoter of safflower MYBs. Figure S7: Expression of MYBs in safflowers at different flowering stages, under different light intensity treatments and different tissues. Figure S8: Expression clusters of MYBs in safflower at different flowering stages, tissues and under different light intensity treatments. Genes were categorized into six separate clusters. Figure S9: Protein interaction visualization network. Table S1: Comprehensive information of the MYB superfamily in safflowers. Table S2: Number of safflower MYB genes. Table S3: Function and category of each cis-acting element identified in the promoter of safflower. Table S4: Results of gene clustering analysis.

## Abbreviations

*C.tinctorius*	*Carthamus tinctorius L*
*A. thaliana*	*Arabidopsis thaliana*
*O. sativa*	*Oryza sativa*
HYSA	Hydroxysafflor yellow A
UGT	UDP-glycosyltransferase
OGT	O-linked N-acetylglucosamine (GlcNAc) transferase
MeJA	Methyl jasmonate
bHLH	Basic helix–loop–helix
CHS	Chalcone synthase
F3H	Flavanone 3-hydroxylase
FPKM	Fragments per kilobase of transcript per million mapped reads
CDS	Coding sequences
UTR	Untranslatted regions
qPCR	Quantitative real-time PCR
LTR	Long terminal repeat
MBS	MYB binding site
