# Supplementary material for: Genome-Wide Screening for MYB Transcription Factors Involved in Flavonoid Glycoside Biosynthesis in Carthamus tinctorius L
Source: Genes (Basel). 2025 Nov 11;16(11):1376. doi: 10.3390/genes16111376 (PMC12652254; doi:10.3390/genes16111376)

Supplementary figure S1

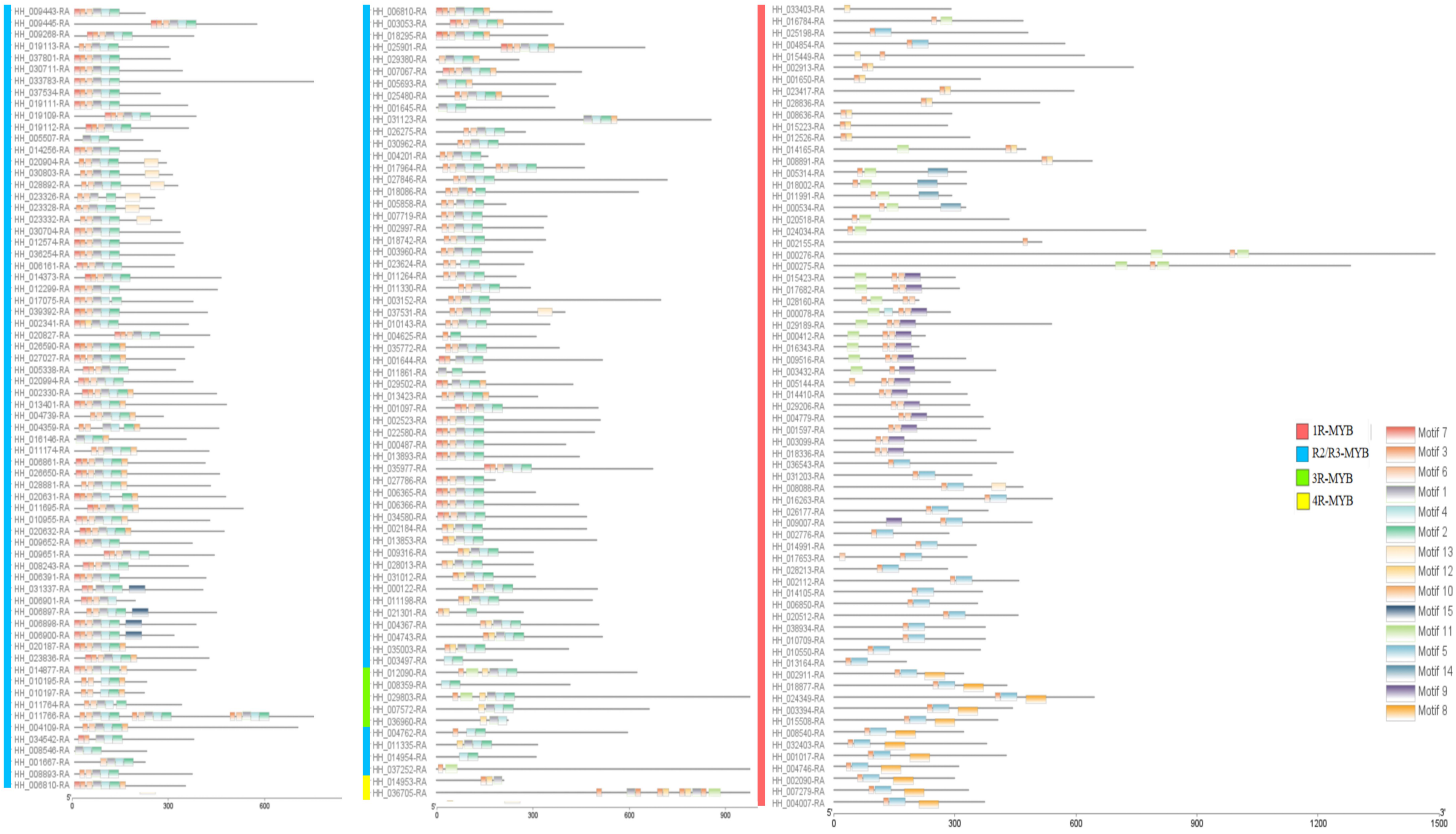

Supplementary figure S2

Motif 1
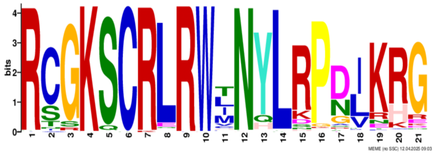

Motif 6
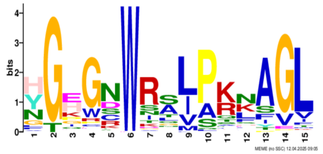

Motif 11
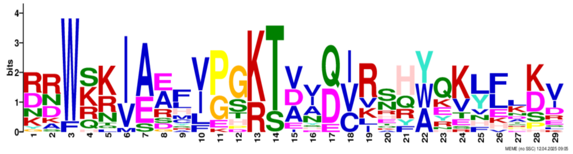

Motif 2
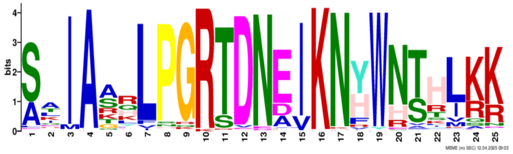

Motif 7
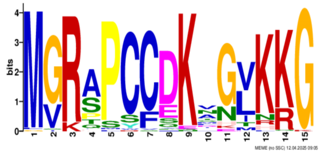

Motif 12
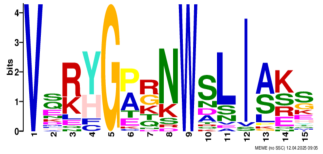

Motif 3
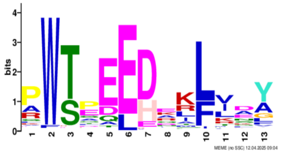

Motif 8
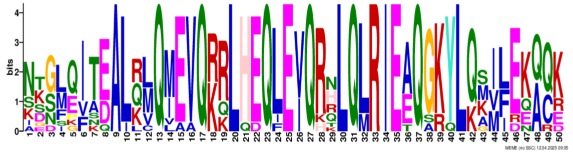

Motif 13
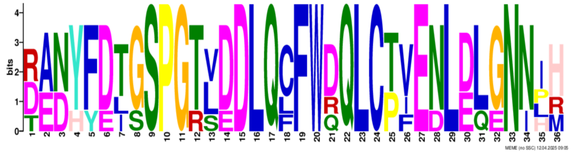

Motif 4
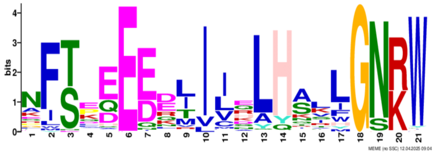

Motif 9
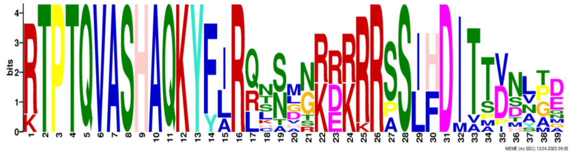

Motif 14
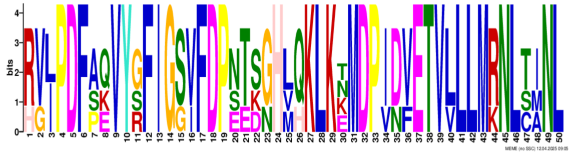

Motif 5
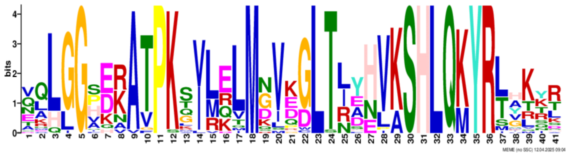

Motif 10
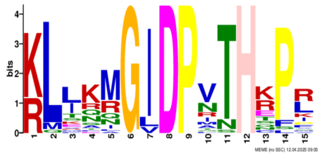

Motif 15
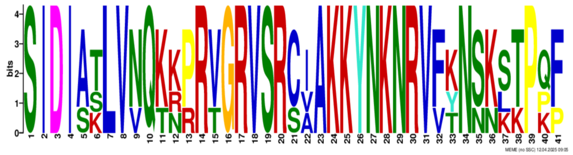

Supplementary figure S3

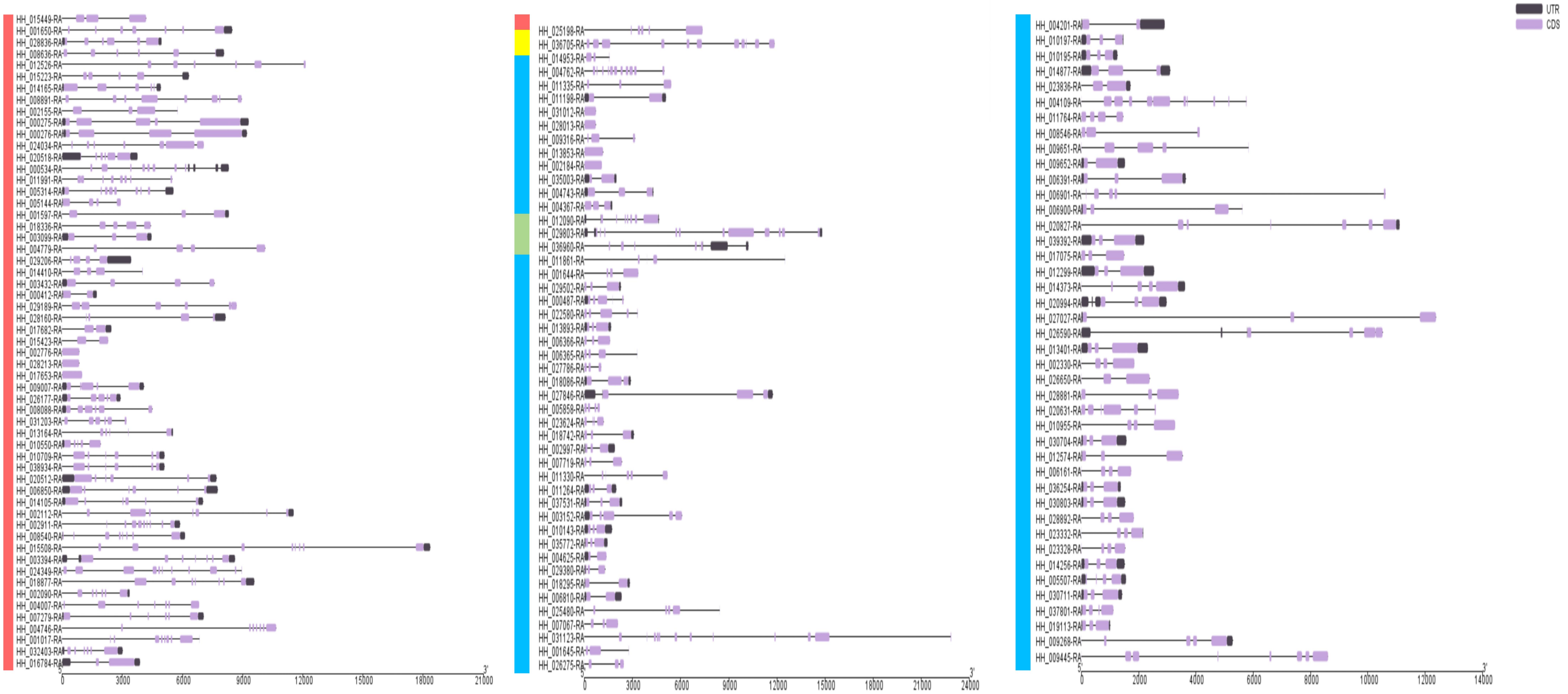

Supplementary figure S4

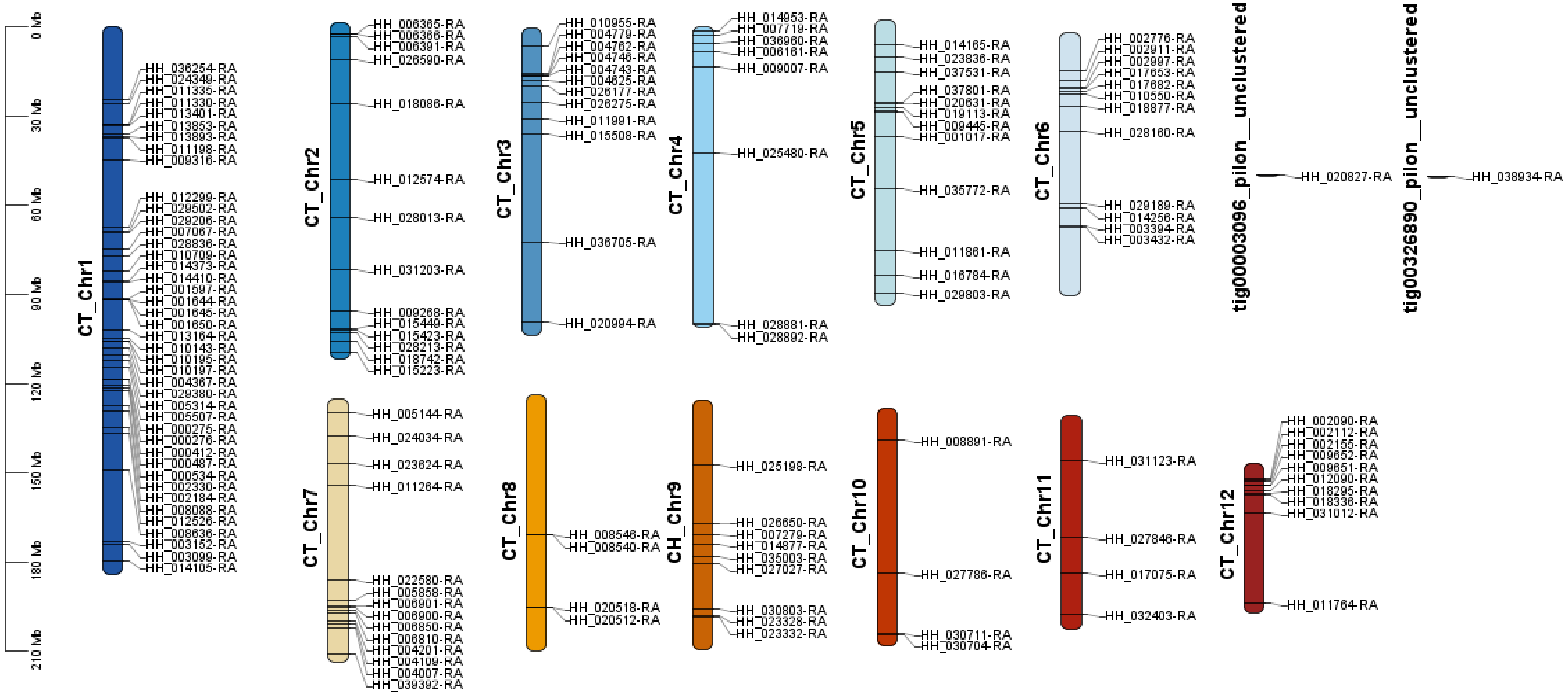

Supplementary figure S5

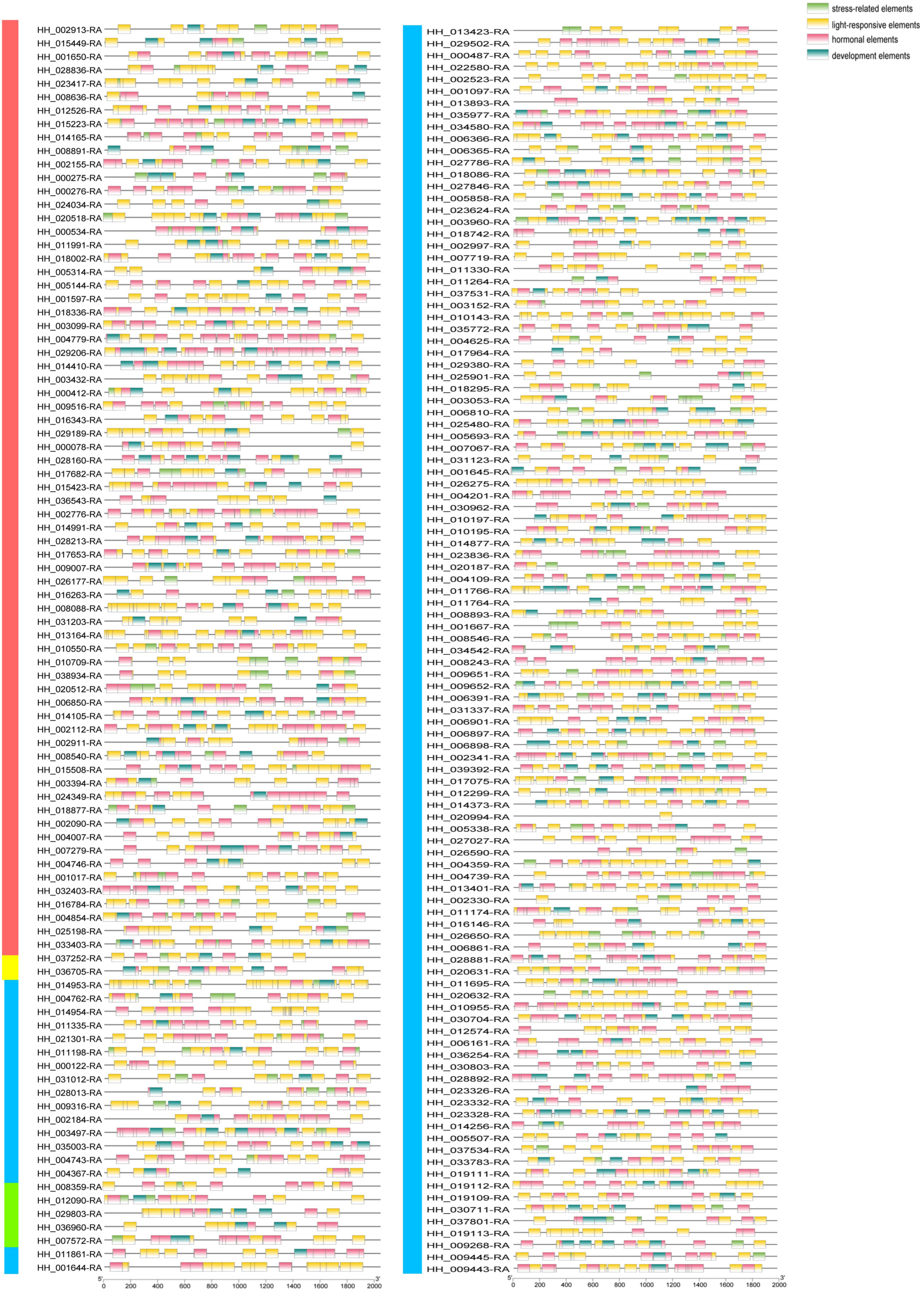

Supplementary figure S6

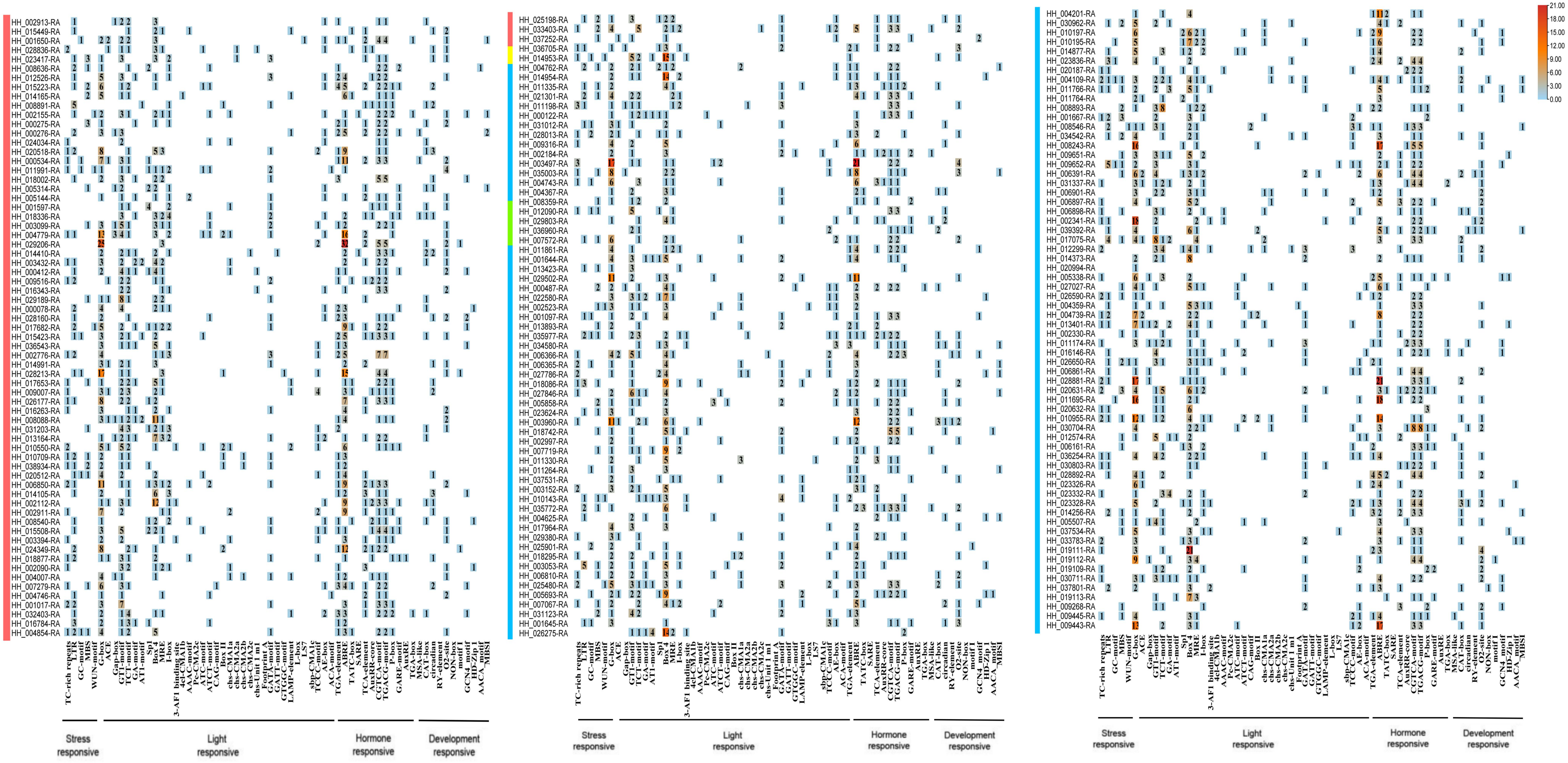

Supplementary figure S7

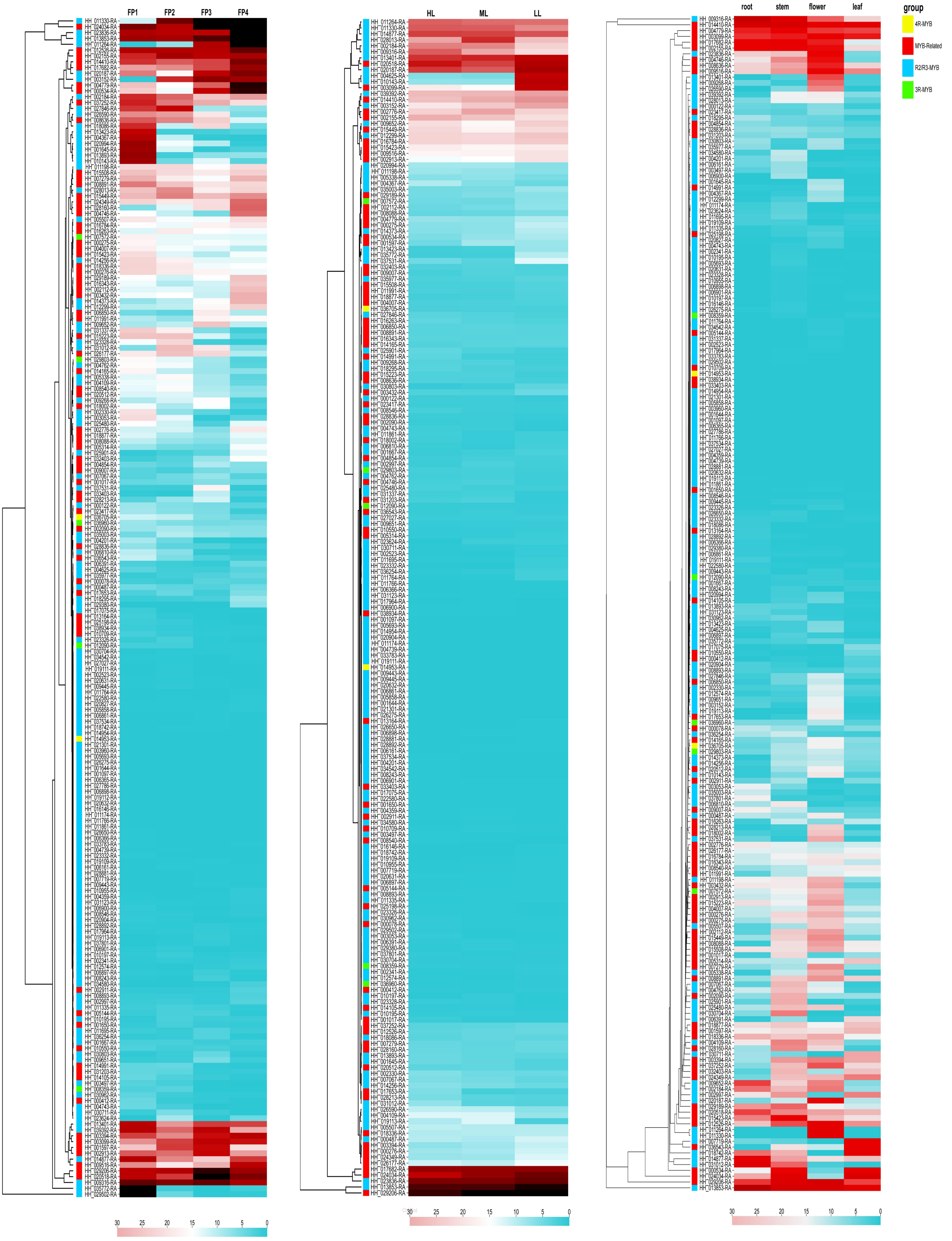

Supplementary figure S8

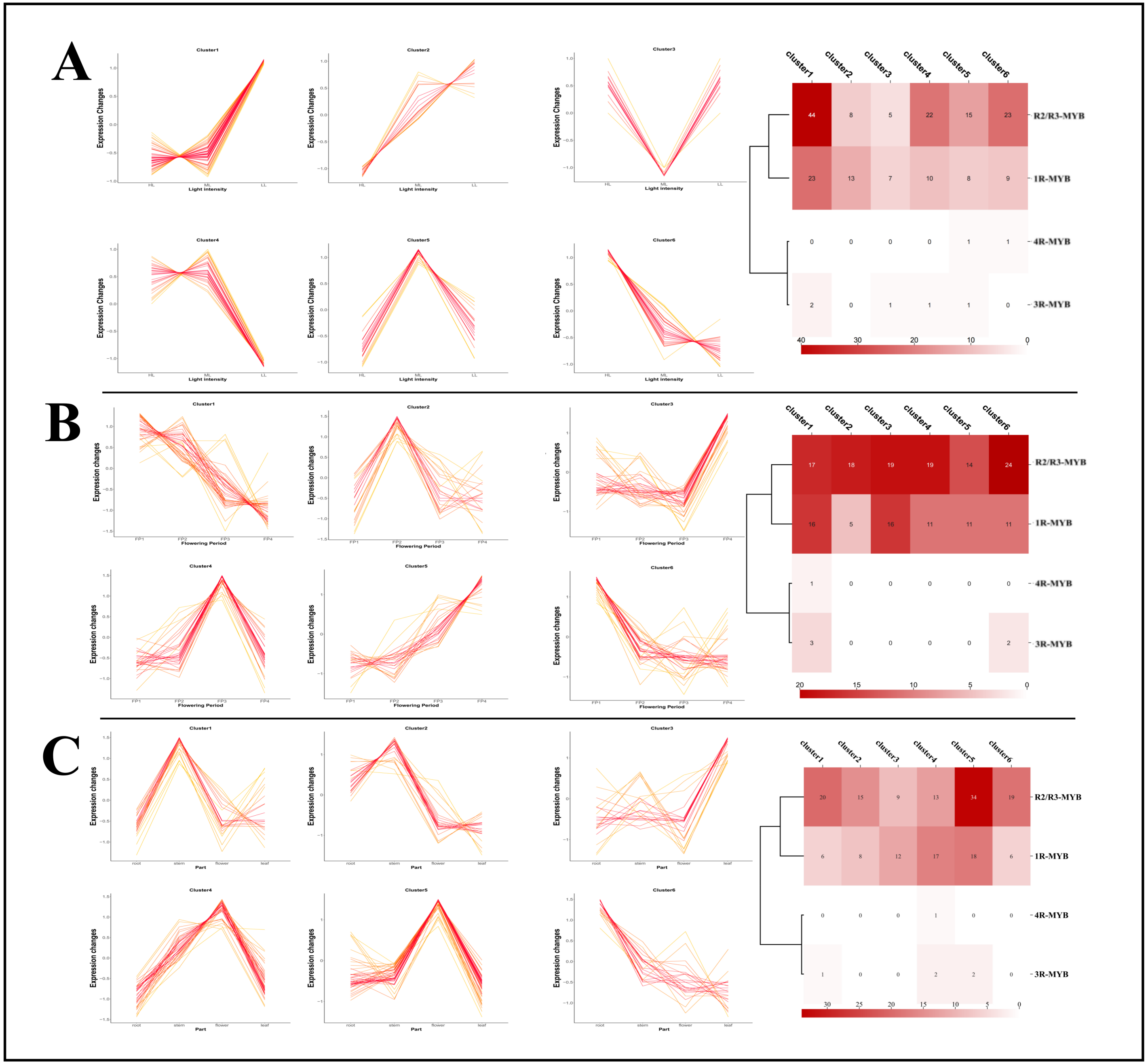

Supplementary figure S9

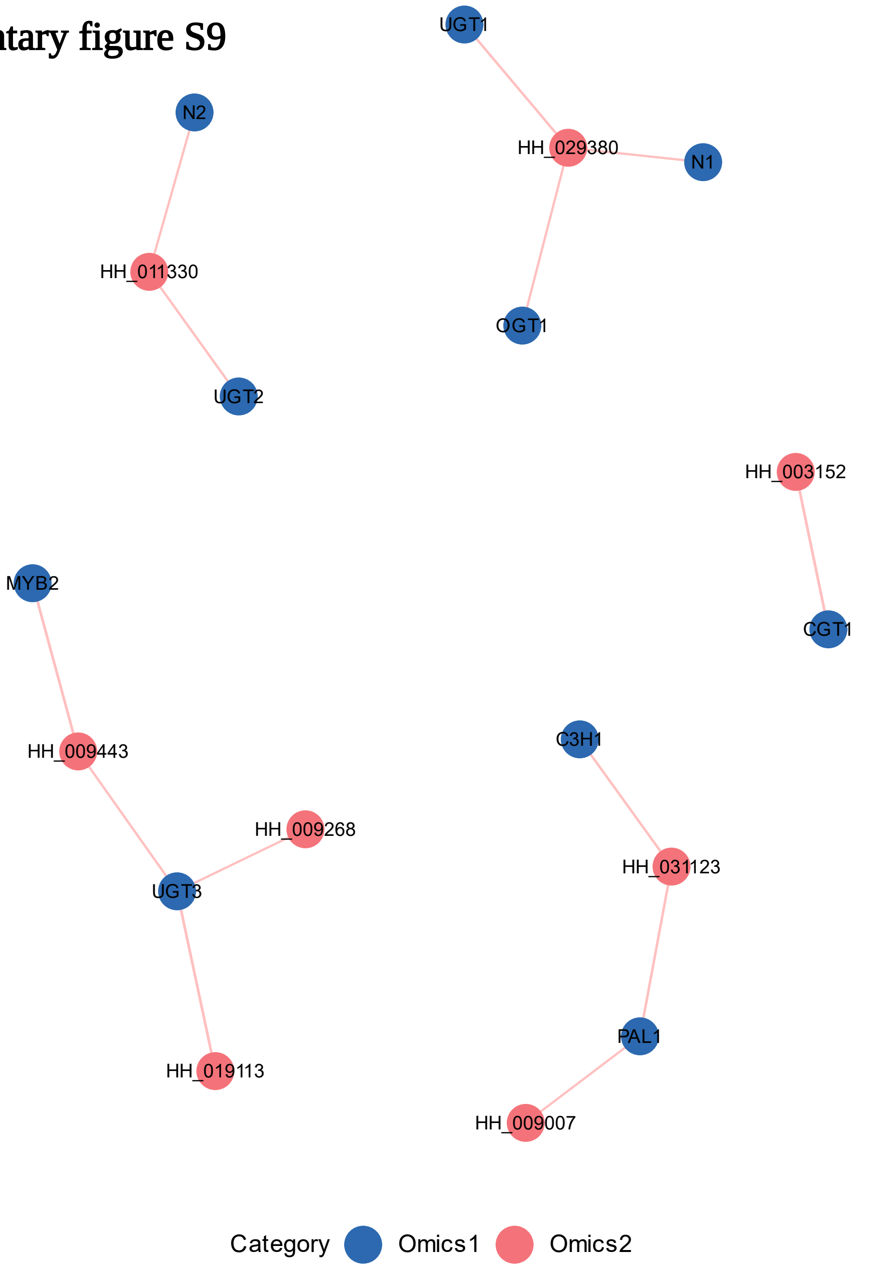

Supplement: Supplementary file 1 [file genes-16-01376-s001.zip › Supplementary Figures.pdf]
